# Cardiac injury associated with severe disease or ICU admission and death in hospitalized patients with COVID-19: a meta-analysis and systematic review

**DOI:** 10.1186/s13054-020-03183-z

**Published:** 2020-07-28

**Authors:** Xinye Li, Xiandu Pan, Yanda Li, Na An, Yanfen Xing, Fan Yang, Li Tian, Jiahao Sun, Yonghong Gao, Hongcai Shang, Yanwei Xing

**Affiliations:** 1 Guang’anmen Hospital, China Academy of Chinese Medical Sciences, Beijing, China; 2 Beijing University of Chinese Medicine, Beijing, China; 3 Institute of Basic Research In Clinical Medicine, China Academy Of Chinese Medical Sciences, Beijing, China; 4 Key Laboratory of Chinese Internal Medicine of the Ministry of Education, Dongzhimen Hospital Affiliated to Beijing University of Chinese Medicine, Beijing, China; 5 Shanxi University of Chinese Medicine, Taiyuan, China

**Keywords:** Cardiac injury, Biomarkers, COVID-19, Meta-analysis, Mortality

## Abstract

**Background:**

Cardiac injury is now a common complication of coronavirus disease (COVID-19), but it remains unclear whether cardiac injury-related biomarkers can be independent predictors of mortality and severe disease development or intensive care unit (ICU) admission.

**Methods:**

Two investigators searched the PubMed, EMBASE, Cochrane Library, MEDLINE, Chinese National Knowledge Infrastructure (CNKI), Wanfang, MedRxiv, and ChinaXiv databases for articles published through March 30, 2020. Retrospective studies assessing the relationship between the prognosis of COVID-19 patients and levels of troponin I (TnI) and other cardiac injury biomarkers (creatine kinase [CK], CK myocardial band [CK-MB], lactate dehydrogenase [LDH], and interleukin-6 [IL-6]) were included. The data were extracted independently by two investigators.

**Results:**

The analysis included 23 studies with 4631 total individuals. The proportions of severe disease, ICU admission, or death among patients with non-elevated TnI (or troponin T [TnT]), and those with elevated TnI (or TnT) were 12.0% and 64.5%, 11.8% and 56.0%, and 8.2% and. 59.3%, respectively. Patients with elevated TnI levels had significantly higher risks of severe disease, ICU admission, and death (RR 5.57, 95% CI 3.04 to 10.22, *P* < 0.001; RR 6.20, 95% CI 2.52 to 15.29, *P* < 0.001; RR 5.64, 95% CI 2.69 to 11.83, *P* < 0.001). Patients with an elevated CK level were at significantly increased risk of severe disease or ICU admission (RR 1.98, 95% CI 1.50 to 2.61, *P* < 0.001). Patients with elevated CK-MB levels were at a higher risk of developing severe disease or requiring ICU admission (RR 3.24, 95% CI 1.66 to 6.34, *P* = 0.001). Patients with newly occurring arrhythmias were at higher risk of developing severe disease or requiring ICU admission (RR 13.09, 95% CI 7.00 to 24.47, *P* < 0.001). An elevated IL-6 level was associated with a higher risk of developing severe disease, requiring ICU admission, or death.

**Conclusions:**

COVID-19 patients with elevated TnI levels are at significantly higher risk of severe disease, ICU admission, and death. Elevated CK, CK-MB, LDH, and IL-6 levels and emerging arrhythmia are associated with the development of severe disease and need for ICU admission, and the mortality is significantly higher in patients with elevated LDH and IL-6 levels.

**Graphical abstract:**

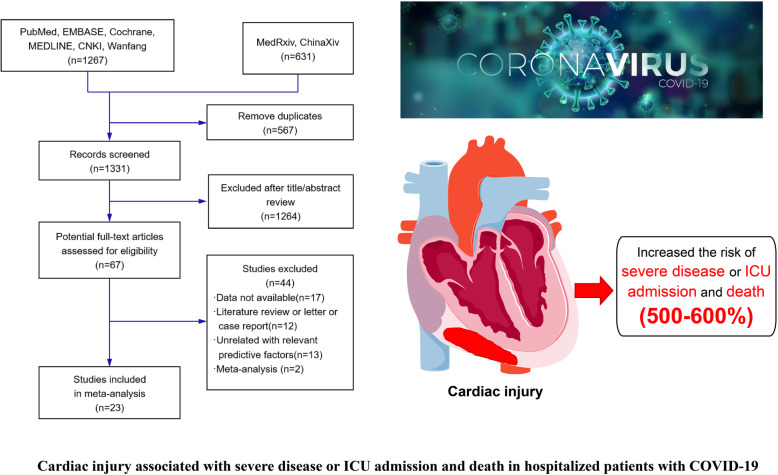

## Background

Coronavirus disease (COVID-19) has spread worldwide, becoming a public health and medical care challenge in many countries. As of April 25, 2020, COVID-19 had spread to 213 countries, areas or territories, with 2,719,897 confirmed cases and 187,705 confirmed deaths worldwide [1]. COVID-19, the clinical manifestation of severe acute respiratory syndrome coronavirus-2 (SARS-CoV-2) infection, is characterized by respiratory tract symptoms. Severe cases can involve acute respiratory distress syndrome (ARDS) and shock [2]. COVID-19 is considered mainly a respiratory tract disease, but cardiovascular complications can also occur, eventually leading to sudden deterioration [3, 4]. A large-scale study including 44,672 patients reported that cardiovascular disease was the risk factors for fatality of COVID-19 patients [5]. Intensive care unit (ICU) occupancy is very fluid, and COVID-19 patients still require better evidence-based cardiovascular treatment [6]. Inciardi et al. reported the case of a patient who recovered from the influenza-like syndrome but then developed symptoms of heart failure [3]. A recent study recommended that cardiac biomarkers should be evaluated in all hospitalized patients with confirmed COVID-19 [7]. However, there has been less concern about cardiac complications in other published studies. Data such as those from transthoracic echocardiography, cardiac magnetic resonance imaging (MRI), coronary angiography, and other examinations of cardiovascular diseases, as well as the biomarkers of cardiac injury have been less often described or are even missing.

Recent case reports have suggested that acute cardiac injury can cause cardiac dysfunction, leading to cardiogenic shock and the proclivity for malignant arrhythmia [8]. Another study reported that COVID-19 was associated with myocarditis and arrhythmia [9]. Studies have shown that cardiac injury is related to higher in-hospital mortality rate [4] and is commonly observed in severe COVID-19 cases [9]. Therefore, paying attention to the occurrence of cardiac complications in patients with COVID-19 and performing risk stratification may greatly reduce patient mortality rates, especially of those with severe disease or requiring ICU admission. To our knowledge, this is the first study to comprehensively evaluate the impact of cardiac injury and its related biomarkers on mortality and other prognosis in patients infected with SARS-CoV-2.

## Methods

### Data sources and study selection

This meta-analysis was performed according to the Preferred Reporting Items for Systematic Reviews and Meta-analysis statement [10]. Two investigators (X.L. and Y.X.) independently conducted a comprehensive search of the relevant literature published until March 30, 2020, in the PubMed, EMBASE, Cochrane Library, MEDLINE, Chinese National Knowledge Infrastructure (CNKI), Wanfang, MedRxiv, and ChinaXiv databases. Combinations of the relevant medical subject heading (MeSH) terms, key words, and word variants of “novel coronavirus,” “coronavirus disease 2019,” “COVID-19,” “2019-nCoV,” “SARS-2-CoV,” “clinical or characteristic,” and “relative risk or RR” were utilized to identify all potentially relevant studies. After the elimination of duplicates, the titles and abstracts of all retrieved studies were assessed by two independent reviewers (Y.L. and N.A.) to eliminate irrelevant articles. Any disagreements were settled by consensus or by a third reviewer. Language restrictions were not applied during filtering, to maximize search sensitivity.

The inclusion criteria were as follows: (1) diagnosis of COVID-19 according to the World Health Organization interim guidance [11] and (2) reported prognosis (severe disease, ICU admission, or death) with or without cardiac injury, reported cardiac injury biomarkers (for example, troponin I [TnI], troponin T [TnT], creatine kinase [CK], CK myocardial band [CK-MB], and lactate dehydrogenase [LDH]), or arrhythmia. The exclusion criteria were as follows: (1) repeated articles, letters, editorials, and expert opinions and (2) studies with overlapping or unusable data. The primary outcome was the incidence of death, severe disease, or ICU admission in COVID-19 patients with elevated TnI levels versus non-elevated TnI levels. The secondary outcomes were as follows: (1) incidences of elevated TnI, CK, CK-MB, LDH, or interleukin-6 (IL-6) of the non-severe disease/non-ICU versus severe disease/ICU groups; (2) incidences of elevated TnI, CK, CK-MB, LDH, or IL-6 of the survivors versus non-survivors groups; (3) TnI, CK, CK-MB, LDH, or IL-6 levels of the non-severe disease/non-ICU versus severe disease/ICU groups; (4) TnI, CK, CK-MB, LDH, or IL-6 levels of the survivors versus non-survivors groups; (5) incidence of arrhythmia (defined as newly occurring of any type) of the non-severe disease/non-ICU versus severe disease/ICU groups.

### Data extraction

Two investigators (X.L. and X.P.) independently extracted the relevant data from the eligible studies using predesigned forms. Disagreements were resolved by consensus. If the mean and standard deviation (SD) of the laboratory findings were not directly given, we used the estimation formula based on the median, range, and sample size [12]. Definitions used for severity assessment, ICU admission, and cardiac injury were also extracted.

### Quality assessment and publication bias

Two researchers (X.P. and N.A.) independently assessed the quality of the included studies, using the Newcastle-Ottawa Quality Assessment Scale [13]. Studies were defined as high quality if a score of 7 or higher was attained [13]. Potential publication bias was evaluated using the visual inspection of funnel plots and formal testing with the Egger’s testing [14].

### Statistical analysis

Effect estimates are presented as relative risk (RR) or standard mean differences (SMD) with 95% confidence interval (CI). The *I*^*2*^ statistic was used to quantify the heterogeneity across studies. *I*^*2*^ > 50% suggested significant statistical heterogeneity [15]. In this case, a random-effects model was used considering the intra- and interstudy variation. Otherwise, the pooled effect was calculated using a fixed-effects model. All analyses were performed using Stata 16.0 (StataCorp, College Station, TX, USA). Values of *P* < 0.05 were considered statistically significant.

## Results

### Study selection

**Fig. 1:**
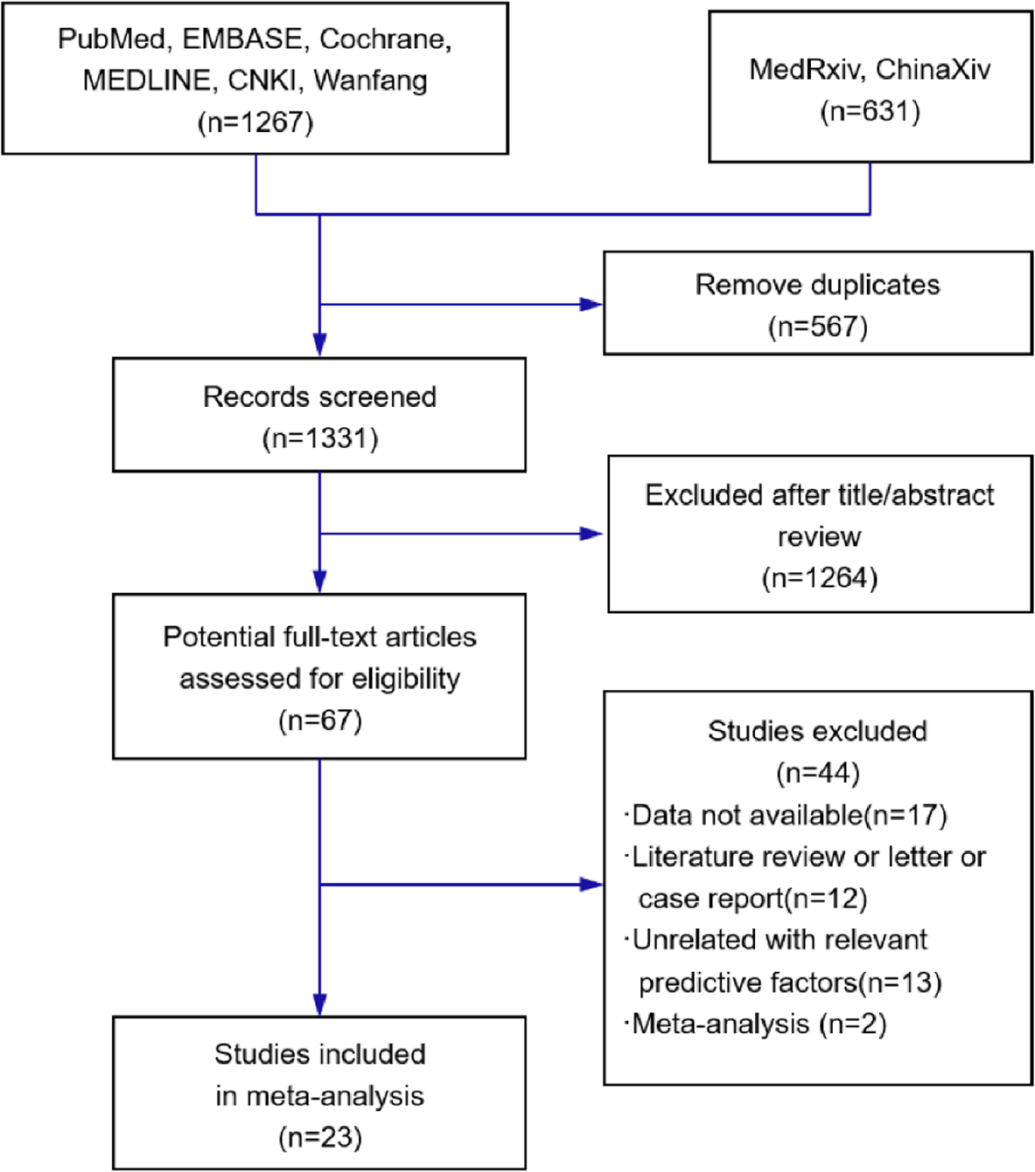
Flow diagram showing study search and selection. CNKI=Chinese National Knowledge Infrastructure

We identified 1898 studies using the predefined search terms. After the removal of duplicates and filtering of titles and abstracts to exclude irrelevant articles, 67 records remained. The full text of the 67 records was reviewed; of them, 44 records were excluded for the following reasons: data not available (*n* = 17), literature review or letter or case report (*n* = 12), unrelated to relevant predictive factors (*n* = 13), and meta-analysis (*n* = 2). Finally, 23 studies were included in this meta-analysis, of which one was not written in English. The flow diagram of this study selection is shown in Fig. 1.

### Study characteristics

The primary characteristics of the 23 included studies are listed in Table 1, with 4631 individuals incorporated. The sample size of 16 studies was greater than 100. The definition of cardiac injury was extracted (Table 2). The clinical characteristics of all included patients with COVID-19 are shown in Additional file 1: Table S1. Overall, 16 studies reported cardiac injury biomarkers, and 4 reported arrhythmias. All the results calculated using Stata are shown in Table 3.

**Table 1:** Characteristics of the studies included in the meta-analysis and systematic review

Study	Study period and location	Study design	Population, ***N***	Male, ***N*** (%)	Median/mean age, year	Non-severe disease/severe disease, ***N***	Non-ICU/ICU, ***N***	Survivors/Non-survivors, ***N***	Definition of severe disease	Study population	Quality score
Wang DW et al. [16]	Jan 1 to Jan 28, 2020, Wuhan, China	SC, retrospective case series	138	75 (54.3)	56 (IQR: 42–68)	NA	102/36	NA	Developed ARDS	Consecutive hospitalized patients with confirmed NCIP	9
Wu CM et al. (a) [17]	Dec 25, 2019, to Jan 26, 2020, Wuhan, China	SC, retrospective cohort study	201*	128 (63.7)	51 (IQR: 43–60)	117/84	NA	40/44	Developed ARDS	Patients with confirmed COVID-19 pneumonia	9
Yang XB et al. [18]	Dec 24, 2019, to Jan 26, 2020, Wuhan, China	SC, retrospective, observational study	52	35 (67.3)	59.7 (SD: 13.3)	NA	NA	20/32	NA	Critically ill patients with SARS-CoV-2 pneumonia	8
Huang CL et al. [2]	Dec 16, 2019, to Jan 2, 2020, Wuhan, China	NA, retrospective	41	30 (73.2)	49 (IQR: 41–58)	NA	28/13	35/6	Required high-flow nasal cannula or higher-level oxygen support measures to correct hypoxemia	Patients identified as having laboratory-confirmed 2019-nCoV infection and admitted hospital	8
Chen D et al. [19]	Jan 11 to Feb 15, 2020, Wenzhou, China,	MC, retrospective study	175	83 (47.4)	46 (IQR: 34–54)	40/135	NA	NA	Showed pneumonia and any of acute respiratory distress syndrome	Patients with COVID-19	8
Fu L et al. [20]	Jan 1 to Jan 30,2020, Wuhan, China	SC, retrospective cohort study	200	99 (49.5)	NA	NA	NA	166/34	NA	Patients with confirmed COVID-19	8
Guan WJ et al. [21]	Jan 1 to Jan 29,2020, China	MC, retrospective study	1099	640 (58.2)	47 (IQR: 35–58)	926/173	NA	1084/15	NA	Patients with laboratory-confirmed 2019-nCoV ARD	8
Hui H et al. [22]	Jan 21 to Feb 03, 2020, Beijing, China	SC, retrospective study	41	19 (46.3)	47 (IQR: 35.5–64)	34/7	NA	NA	NA	Patients with confirmed COVID-19	7
Liu YL et al. [23]	Jan 2 to Feb 12, 2020, Wuhan, China	SC, retrospective study	109	59 (54.1)	55 (IQR: 43–66)	56/53	NA	78/31	Developed ARDS	Patients with confirmed COVID-19	8
Liu L et al. [24]	Jan 20 to Feb 3, 2020, Chongqing, China	SC, retrospective case series	51	32 (62.7)	45 (IQR: 34–51)	44/7	NA	NA	NA	Patients with confirmed COVID-19	8
Qi D et al. [25]	Jan 19 to Feb 16, 2020, Chongqing, China	MC, retrospective, descriptive study	267	149 (55.8)	48 (IQR: 35–65)	217/50	214/53	263/4	According to the American Thoracic Society guideline	Patients with COVID-19 confirmed by real-time RT-PCR	7
Wang YF et al. [26]	Jan 1 to Feb 10, 2020, Wuhan, China	SC, retrospective	110	48 (43.6)	NA	72/38	NA	NA	Fever or suspected respiratory infection, plus one of a respiratory rate > 30 breaths/min, severe respiratory distress, or SpO2 < 90% on room air	Patients with confirmed COVID-19 pneumonia	8
Wu CM et al. (b) [27]	Dec 25, 2019 to Jan 27, 2020, Wuhan, China	SC, retrospective cohort study	188	119 (63.3)	51.9 (SD: 14.26)	NA	138/50	145/4	NA	Patients with confirmed COVID-19 pneumonia	8
Xu HY et al. [28]	Jan 02 to Feb 14, 2020, NA	NA, retrospective	53	28 (52.8)	NA	34/19	45/8	53/3	More likely to have underlying comorbidities, and AMI	Consecutive laboratory-confirmed and hospitalized patients with confirmed NCIP	7
Xu YH et al. [29]	Jan 14 to Feb 28, 2020, Guangdong, China	MC, retrospective, observational study	45	29 (64.4)	56.7 (SD: 15.4)	25/20	0/45	44/1	Defined as those required oxygen therapy, symptoms of respiratory distress or required mechanical ventilation	critically ill patients with SARS-CoV-2 pneumonia RT-PCR confirmed positive patients	8
Liu YB et al. [30]	Jan 10 to Feb 24, 2020, Guangzhou, China	SC, retrospective	291	133 (45.7)	48.1 (IQR: 34–62)	262/29	265/26	290/1	NA	Laboratory-confirmed patients with NCIP	8
Peng YD et al. [31]	Jan 20 to Feb, 15, 2020, Wuhan, China	SC, retrospective, cohort study	112	53 (47.0)	62 (IQR: 55–67)	96/16	NA	84/28	Required mechanical ventilation; shock; combined with other organ failure	COVID-19 patients with CVD	7
Zhang GQ et al. [32]	Jan 2 to Feb, 10, 2020, Wuhan, China	SC, retrospective case series study	221	108 (48.9)	55 (IQR: 39–66.5)	166/55	NA	209/12	Fever plus one of these conditions, including respiratory rate ≥ 30 breaths/min, severe respiratory distress, SpO2 ≤ 93% on room air, occurrence of respiratory failure requiring mechanical ventilation, shock and other organ failure	Patients who were confirmed diagnosed as COVID-19 according to WHO interim guidance	8
Liu T et al. [33]	Jan 21, to Feb 16, 2020, Wuhan, China	NA, retrospective	80	34 (42.5)	53 (range: 26–86)	11/69	NA	80/0	Defined when any of the following criteria was met: dyspnea, respiration rate ≥ 30 times/min; oxygen saturation by pulse oximeter ≤93% in resting state; partial pressure of arterial oxygen to fraction of inspired oxygen ratio ≤ 300 mmHg	SARS-CoV-2 nucleic acid or RT-PCR confirmed positive patients	8
Shi SB et al. [4]	Jan 20, to Feb 10, 2020, Wuhan, China	SC, retrospective cohort study	416	205 (49.3)	64 (range: 21–95)	319/97	NA	359/57	NA	Consecutive inpatients with laboratory-confirmed COVID-19	9
Wu J et al. [34]	Jan 20 to Feb 20,2020, Jiangsu and Anhui Province, China	MC, retrospective case series	280	151 (53.9)	43.1 (SD:19.02)	197/83	NA	NA	NA	Patients infected with SARS-CoV-2	8
Chen T et al. [35]	Jan 13, to Feb 28, 2020, Wuhan, China	SC, retrospective	274	171 (62)	62 (IQR: 44–70)	NA	NA	161/113	NA	Patients with confirmed COVID-19 pneumonia	8
Guo T et al. [36]	Jan 23, to Feb 23, 2020, Wuhan, China	SC, retrospective	187	91 (48.7)	58.5 (SD:14.66)	NA	NA	144/43	NA	Patients with confirmed COVID-19 pneumonia	9

*AMI* acute myocardial injury, *ARDS* acute respiratory distress syndrome, *COVID-19* coronavirus disease 2019, *CVD* cardiovascular disease, *2019-nCoV* novel coronavirus, *IQR* interquartile range, *MC* multicenter study, *N* number, *NA* not available, *NCIP* novel coronavirus-infected pneumonia, *RT-PCR* reverse transcriptase polymerase chain reaction, *SARS-CoV-2* severe acute respiratory syndrome coronavirus-2, *SC* single-center study, *SD* standard deviation

**Table 2:** Clinical characteristics of COVID-19 patients with or without elevated TnI (TnT)

Source	Severe disease/ARDS		ICU		Death		NT-proBNP, Median (IQR), pg/mL		Definition of cardiac injury
	Elevated TnI/TnT, ***N***/total	Non-elevated TnI/TnT, ***N***/total	Elevated TnI/TnT, ***N***/total	Non-elevated TnI/TnT, ***N***/total	Elevated TnI/TnT, ***N***/total	Non-elevated TnI/TnT, ***N***/total	Elevated TnI/TnT	Non-elevated TnI/TnT	
Liu et al. [30]	11/15	18/276	11/15	15/276	1/15	0/276	NA	NA	TnI > 0.03 μg/L
Xu et al. [28]	6/6	13/47	6/6	2/47	3/6	0/47	NA	NA	TnT-HSST > 28 pg/ml
Wu et al. (b) [27]	NA	NA	27/62	23/126	31/62	12/126	NA	NA	Hs-TnI ≥ 6.126 pg/mL
Hui et al. [22]	4/4	1/16	NA	NA	NA	NA	NA	NA	NA
Wang et al. [16]	NA	NA	8/10	28/128	NA	NA	NA	NA	NA
Yang et al. [18]	NA	NA	NA	NA	9/12	23/40	NA	NA	Hs-TNI > 28 pg/mL
Shi et al. [4]	48/82	49/334	NA	NA	42/82	15/334	1689 (698–3327)	139 (51–335)	Hs-TnI > 0.04 ng/mL
Chen et al. [35]	NA	NA	NA	NA	68/83	26/120	NA	NA	Hs-TnI > 15.6 pg/mL
Guo et al. [36]	NA	NA	NA	NA	31/52	12/135	817.4 (336.0–1944.0)	141.4 (39.3–303.6)	Elevated TnT levels

*ARDS* acute respiratory distress syndrome, *Hs-TnI* high-sensitivity troponin I, *ICU* intensive care unit, *IQR* interquartile range, *N* number, *NA* not available, *NT-proBNP* N-terminal pro-B-type natriuretic peptide, *TnI* troponin I, *TnT* troponin T, *TnT-HSST* troponin T-hypersensitivity

**Table 3:** All the results calculated using Stata

Characteristic	Non-elevated TnI/TnT vs. elevated TnI/TnT					Non-severe disease/non-ICU vs. severe disease/ICU					Survivors vs. non-survivors				
	***I***^***2***^(%)	RR	95%CI	***Z***	***P***	***I***^***2***^(%)	RR/SMD	95%CI	***Z***	***P***	***I***^***2***^(%)	RR/SMD	95%CI	***Z***	***P***
Severe, *n*	78.6	5.57	(3.04, 10.22)	5.55	0.00	NA	NA	NA	NA	NA	NA	NA	NA	NA	NA
ICU, *n*	89.3	6.20	(2.52, 15.29)	3.96	0.00	NA	NA	NA	NA	NA	NA	NA	NA	NA	NA
Death, *n*	89.1	5.64	(2.69, 11.83)	4.57	0.00	NA	NA	NA	NA	NA	NA	NA	NA	NA	NA
NT-proBNP (pg/ml)	86.6	1.63	(1.02, 2.23)	5.27	0.00	NA	NA	NA	NA	NA	NA	NA	NA	NA	NA
Elevated TnI/TnT, *n*	NA	NA	NA	NA	NA	83.7	15.10	(4.10, 55.61)	4.08	0.00	22.5	4.69	(3.39, 6.48)	9.37	0.00
TnI (pg/ml)	NA	NA	NA	NA	NA	84.6	0.74	(0.19, 1.30)	2.62	0.009	NA	NA	NA	NA	NA
Elevated CK, *n*	NA	NA	NA	NA	NA	0.0	1.98	(1.50, 2.61)	4.78	0.00	NA	NA	NA	NA	NA
CK (U/L)	NA	NA	NA	NA	NA	69.0	0.39	(0.11, 0.67)	2.76	0.006	NA	NA	NA	NA	NA
Elevated CK-MB, *n*	NA	NA	NA	NA	NA	79.8	3.24	(1.66, 6.34)	3.44	0.001	NA	NA	NA	NA	NA
CK-MB (U/L)	NA	NA	NA	NA	NA	NA	NA	NA	NA	NA	NA	NA	NA	NA	NA
Elevated LDH, *n*	NA	NA	NA	NA	NA	79.7	2.20	(1.55, 3.12)	4.40	0.00	NA	NA	NA	NA	NA
LDH (U/L)	NA	NA	NA	NA	NA	92.7	1.15	(0.61, 1.70)	4.16	0.00	98.6	2.86	(0.67, 5.06)	2.56	0.01
BNP (pg/ml)	NA	NA	NA	NA	NA	NA	NA	NA	NA	NA	NA	NA	NA	NA	NA
Arrhythmia, *n*	NA	NA	NA	NA	NA	42.0	13.09	(7.00, 24.47)	8.06	0.00	NA	NA	NA	NA	NA
IL-6 (pg/ml)	NA	NA	NA	NA	NA	0.0	0.54	(0.27, 0.81)	3.94	0.00	13.7	1.28	(1.00, 1.57)	8.85	0.00

*BNP* B-type natriuretic peptide, *CK* creatinine kinase, *CK-MB* creatinine kinase–myocardial band, *ICU* intensive care unit, *LDH* lactate dehydrogenase, *IL-6* interleukin-6, *n* number, *NA* not available, *NT-proBNP* N-terminal pro-B-type natriuretic peptide, *RR* risk ratios, *SMD* standard mean

### Non-elevated TnI vs. elevated TnI groups

**Fig. 2:**
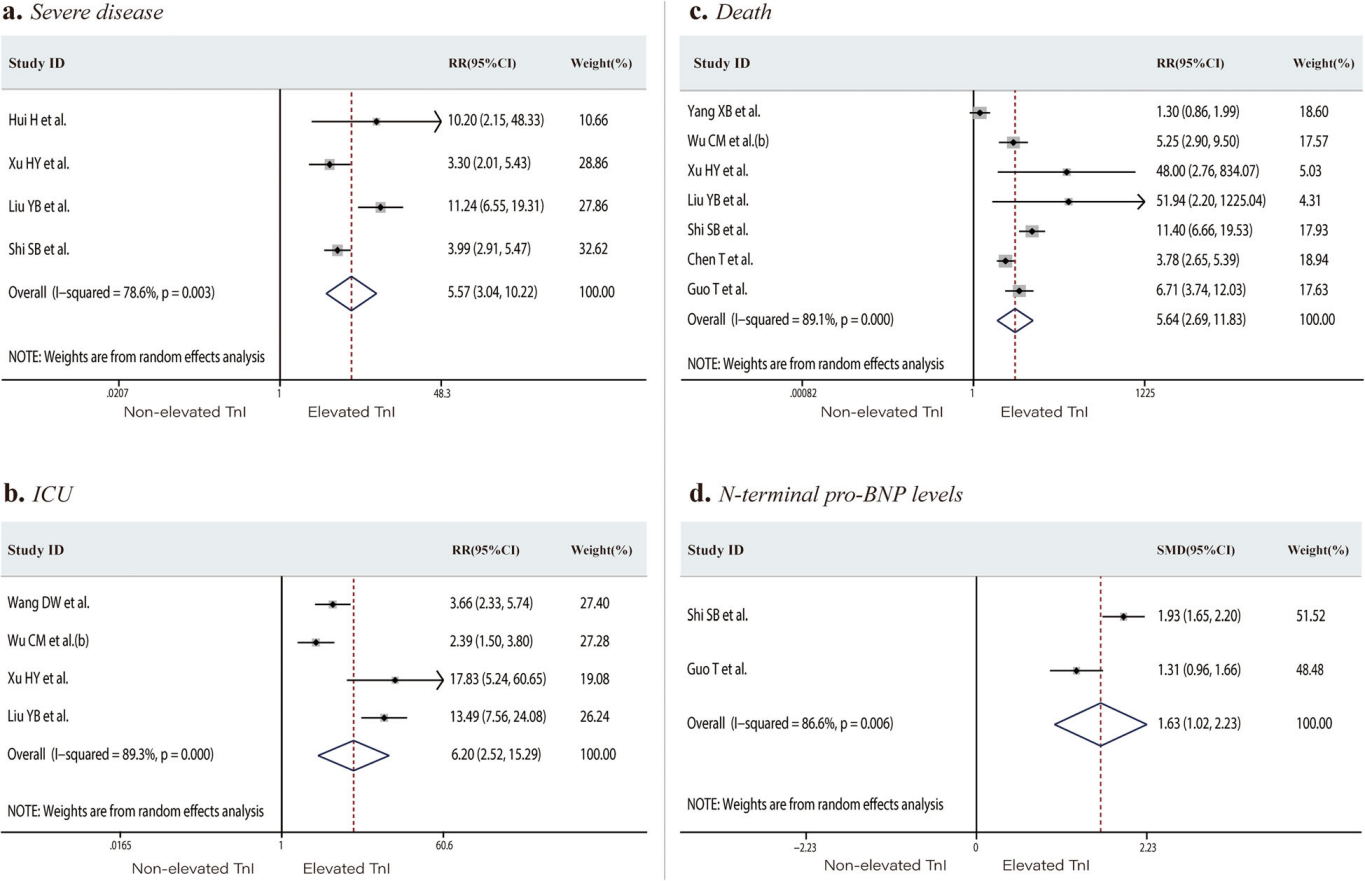
Forest plots comparing of risk of severe disease (**a**), ICU admission (**b**), and death (**c**), and the N-terminal pro-BNP levels (**d**) in patients with or without elevated troponin I (or troponin T). ICU, intensive care unit; BNP, B-type natriuretic peptide; RR, risk ratios; SMD, standard mean

First, we restricted our analysis to studies that assessed the prognosis of COVID-19 patients with or without elevated TnI (or TnT). Nine studies reported the outcome events (including severe disease, ICU admission, and death) of patients with non-elevated or elevated TnI (TnT). TnI, which defines cardiac injury, was measured in a total of 1548 COVID-19 patients. The proportions of severe cases, ICU admissions, or death in the non-elevated TnI versus elevated TnI groups were 12.0% versus 64.5%, 11.8% versus 56.0%, and 8.2% versus 59.3%, respectively. Patients with elevated TnI levels had a significantly higher risk of severe disease, ICU admission, and death than those in the non-elevated TnI group (RR 5.57, 95% CI 3.04 to 10.22, *P* < 0.001; *I*^*2*^ = 78.6%, Fig. 2a; RR 6.20, 95% CI 2.52 to 15.29, *P* < 0.001; *I*^*2*^ = 89.3%, Fig. 2b; RR 5.64, 95% CI 2.69 to 11.83, *P* < 0.001; *I*^*2*^ = 89.1%, Fig. 2c, respectively). The mean N-terminal pro-BNP (NT-proBNP) level was significantly higher in the elevated TnI group than in the non-elevated TnI group (SMD 1.63, 95% CI 1.02 to 2.23, *P* < 0.001; *I*^*2*^ = 86.6%, Fig. 2d).

### Non-severe disease/non-ICU vs. severe disease/ICU groups and survivors vs. non-survivors groups

#### Cardiac injury biomarkers

**Fig. 3:**
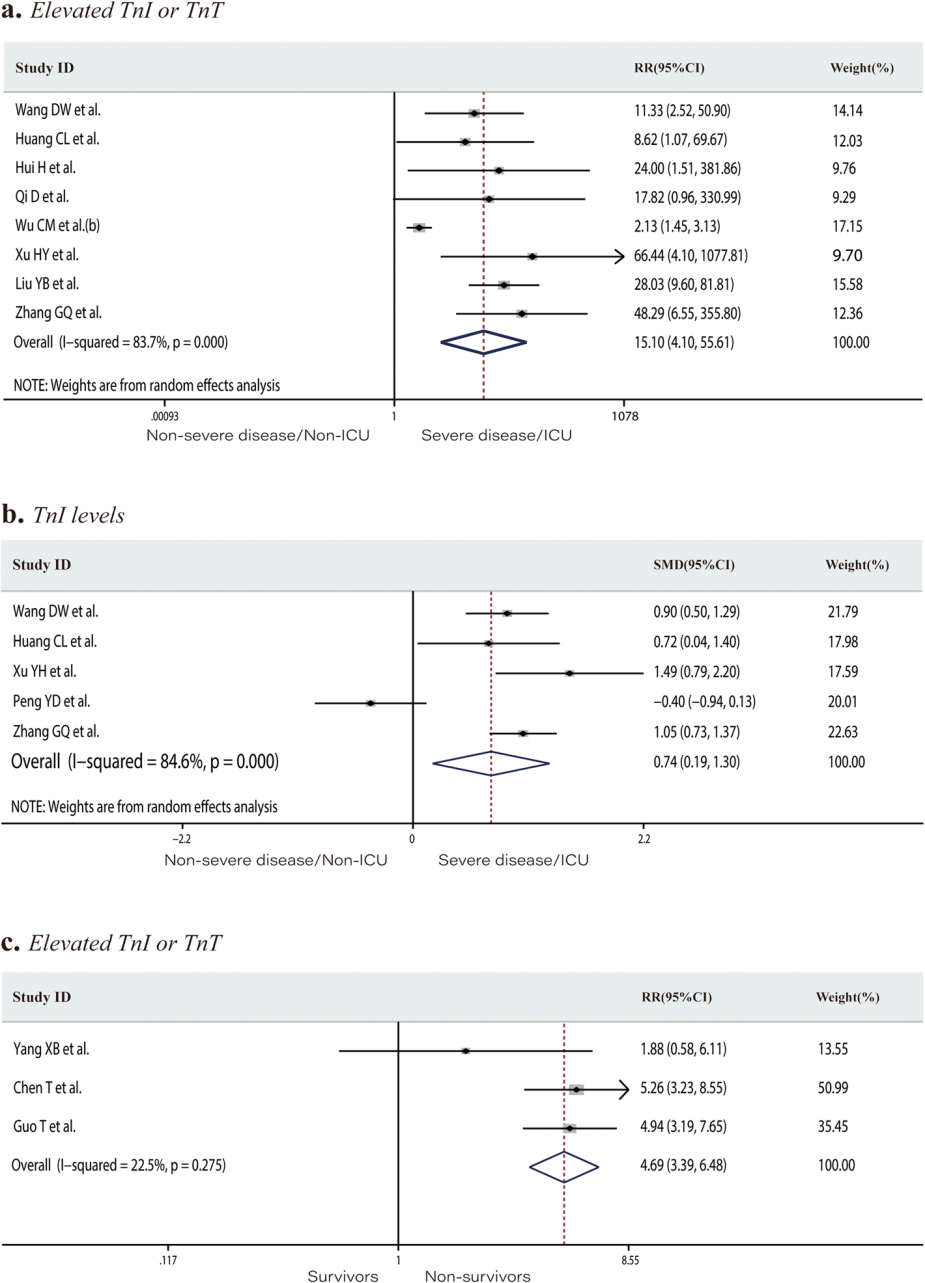
Forest plots comparing of the proportion of patients with elevated troponin I or troponin T levels in the severe disease/ICU group and in the non-severe disease/non-ICU group (**a**), the troponin I levels in the severe disease/ICU group and in the non-severe disease/non-ICU group (**b**), and the proportion of patients with elevated troponin I or troponin T levels in the survivors and non-survivors groups (**c**). ICU, intensive care unit; RR, risk ratios; SMD, standard mean

**Fig. 4:**
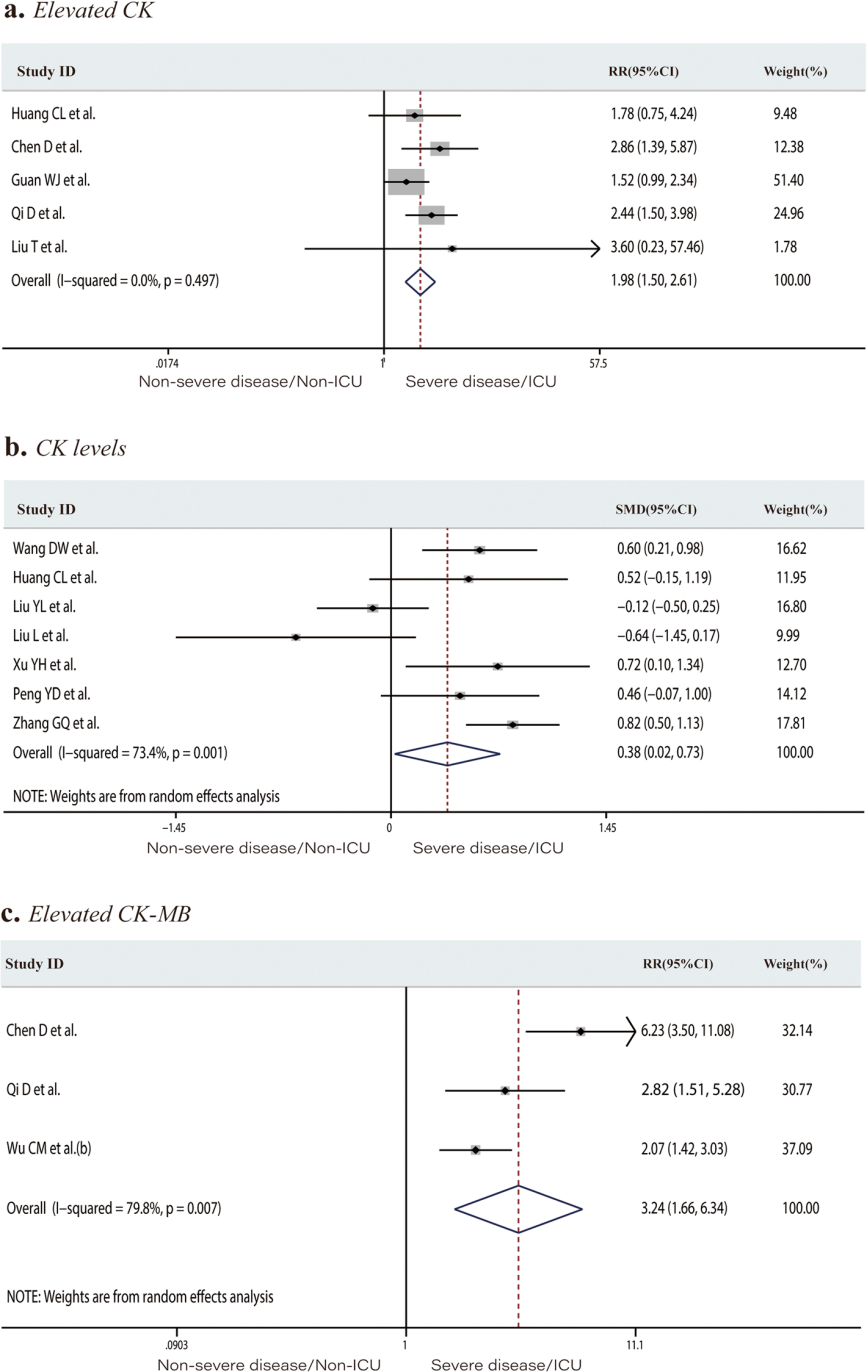
Forest plots comparing of the proportion of patients with elevated creatinine kinase levels in the severe disease/ICU group and in the non-severe disease/non-ICU group (**a**), the creatinine kinase levels in the severe disease/ICU group and in the non-severe disease/non-ICU group (**b**), and the proportion of patients with elevated creatinine kinase–myocardial band levels in the severe disease/ICU group and in the non-severe disease/non-ICU group (**c**). ICU, intensive care unit; RR, risk ratios; SMD, standard mean

**Fig. 5:**
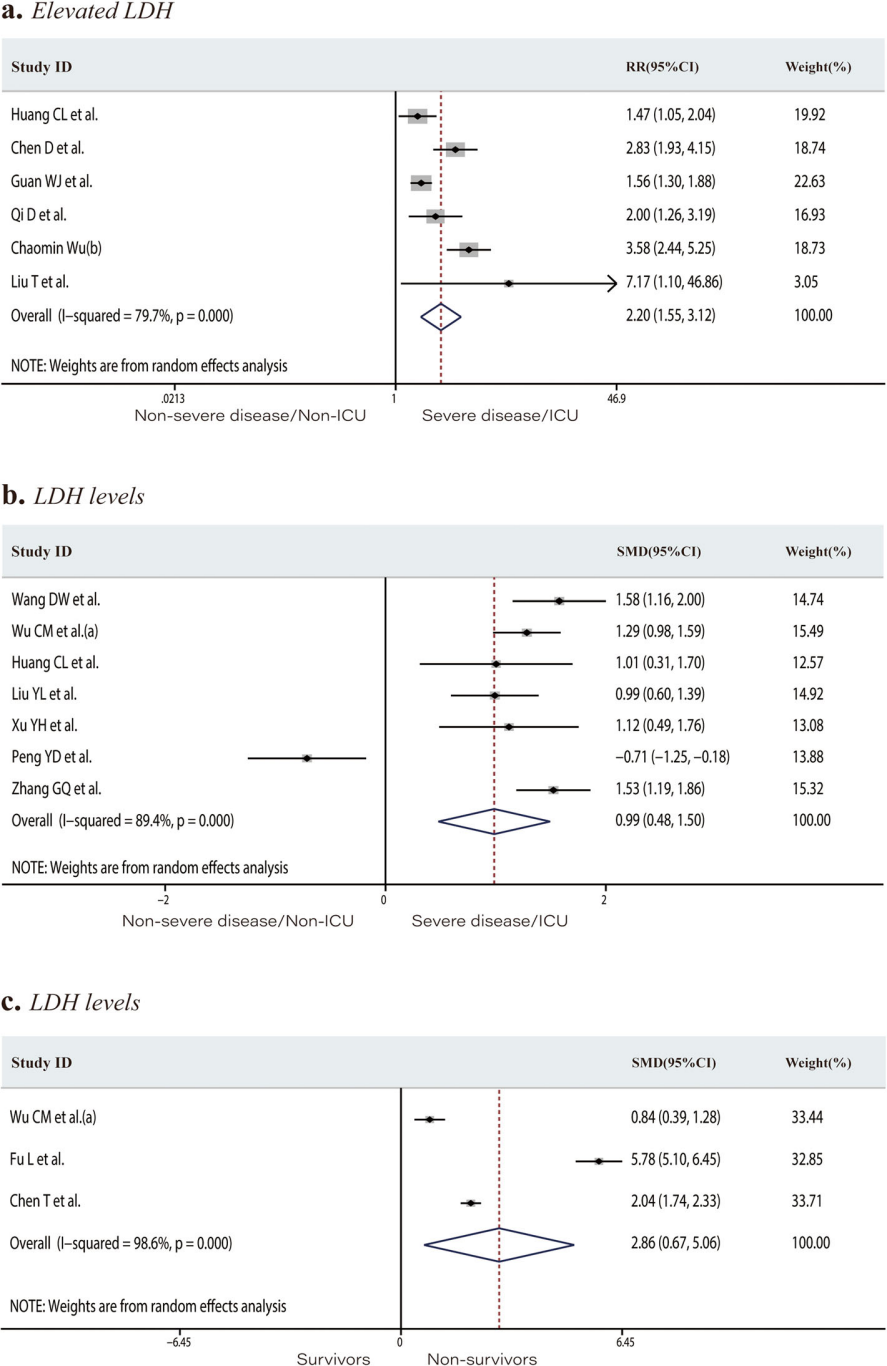
Forest plots comparing of the proportion of patients with elevated lactate dehydrogenase levels in the severe disease/ICU group and in the non-severe disease/non-ICU group (**a**), the lactate dehydrogenase levels in the severe disease/ICU group and in the non-severe disease/non-ICU group (**b**), and the lactate dehydrogenase levels in the survivors and non-survivor groups (**c**). ICU, intensive care unit; RR, risk ratios; SMD, standard mean

Eight studies including 1028 patients in the non-severe disease/non-ICU group or severe disease/ICU group reported the number of patients with elevated TnI or TnT levels (total rate, 11.9%). Elevated TnI or TnT levels occurred at a rate of 2.3% in the non-severe disease/non-ICU group and 36.9% in the severe disease/ICU group. Patients in the severe/ICU group had an increased risk of developing elevated TnI or TnT levels (RR 15.10, 95% CI 4.10 to 55.61, *P* < 0.001; *I*^*2*^ = 83.7%, Fig. 3a). The mean TnI level was significantly higher in the severe disease/ICU admission group (SMD 0.74, 95% CI 0.19 to 1.30, *P* = 0.009; *I*^*2*^ = 84.6%, Fig. 3b). The proportion of patients with elevated TnI or TnT levels in the survivors and non-survivor groups was 14.3% and 63.9%, respectively. Significantly more non-survivors than survivors had elevated TnI or TnT levels (RR 4.69, 95% CI 3.39 to 6.48, *P* < 0.001; *I*^*2*^ = 22.5%, Fig. 3c). Twelve studies including 2174 individuals reported the CK levels or the number of patients with above-normal CK levels. The incidence of elevated CK in the severe disease/ICU group was significantly higher than that in the non-severe disease/non-ICU group (12.9% and 23.2%, respectively; RR 1.98, 95% CI 1.50 to 2.61, *P* < 0.001; *I*^*2*^ = 0.0%, Fig. 4a). The mean CK level was significantly higher in severe disease/ICU group than in the non-severe disease/non-ICU group (SMD 0.39, 95% CI 0.11 to 0.67, *P* = 0.006; *I*^*2*^ = 69.0%, Fig. 4b). The proportion of patients with an elevated CK-MB level in the non-severe disease/non-ICU and severe disease/ICU groups was 14.1% and 45.7%, respectively. Patients in the severe disease/ICU admission group were at higher risk of developing an elevated CK-MB level than those in the non-severe disease/non-ICU group (RR 3.24, 95% CI 1.66 to 6.34, *P* = 0.001; *I*^*2*^ = 79.8%, Fig. 4c). Of the 2532 patients from 15 studies, 29.7% of those in the non-severe disease/non-ICU group versus 60.1% of the severe disease/ICU group had elevated LDH levels. COVID-19 patients with elevated LDH levels were at significantly increased risk of developing severe disease or requiring ICU admission (RR 2.20, 95% CI 1.55 to 3.12, *P* < 0.001; *I*^*2*^ = 79.7%, Fig. 5a). LDH levels were significantly higher in the severe disease/ICU admission group than in the non-severe disease/non-ICU group (SMD 1.15, 95% CI 0.61 to 1.70, *P* < 0.001; *I*^*2*^ = 92.7%, Fig. 5b) and in non-survivors than in survivors (SMD 2.86, 95% CI 0.67 to 5.06, *P* = 0.01; *I*^*2*^ = 98.6%, Fig. 5c).

#### Arrhythmia and IL-6

**Fig. 6:**
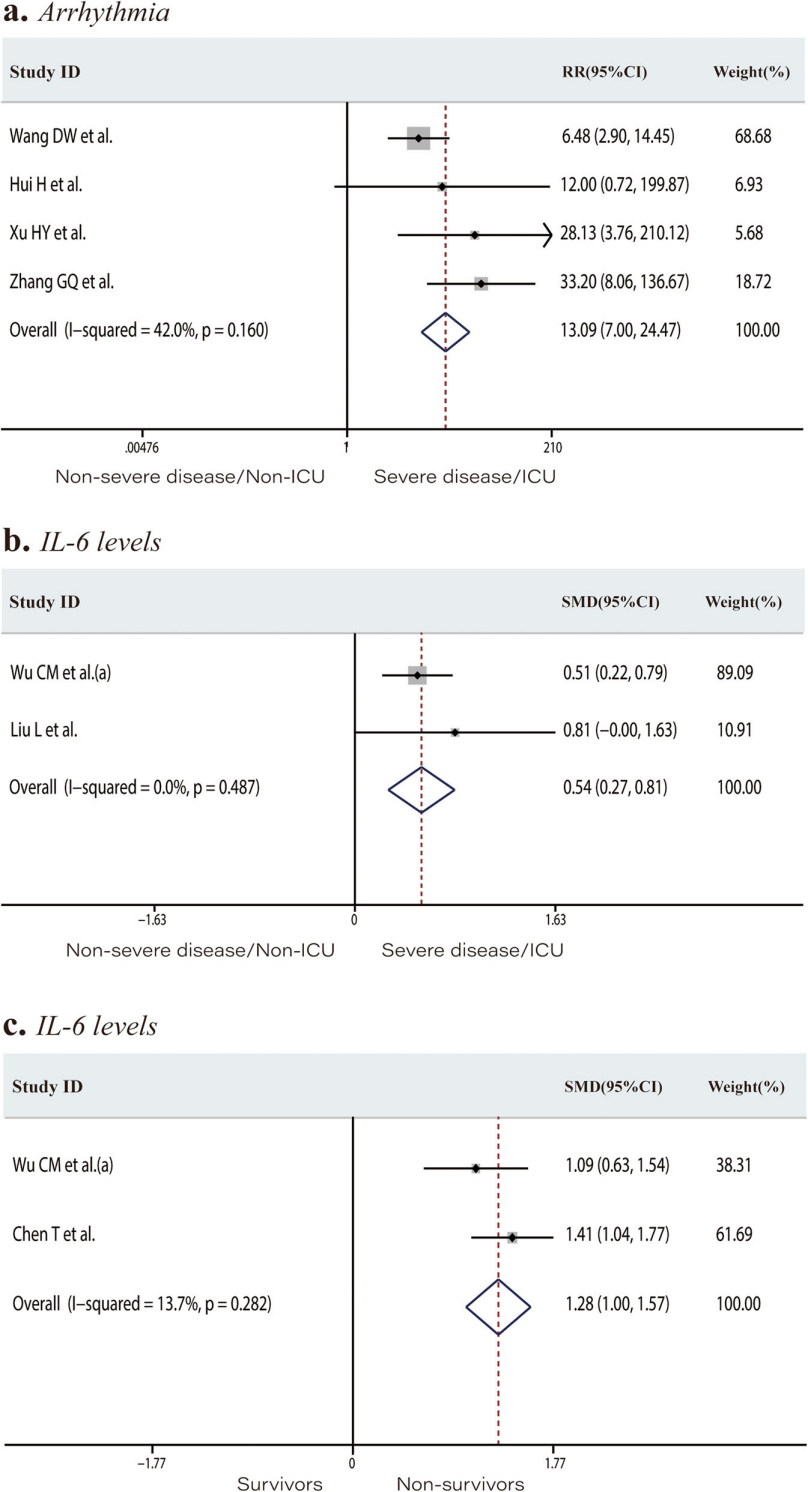
Forest plots comparing of the risk of developing to severe disease or requiring ICU admission among patients with or without newly occurring arrhythmias (**a**), the interleukin-6 levels in the severe disease/ICU group (**b**), and in the non-severe disease/non-ICU group and the interleukin-6 levels in the survivors and non-survivors groups (**c**). ICU, intensive care unit; RR, risk ratios; SMD, standard mean

The incidence of arrhythmia was 3.1% in the non-severe disease/non-ICU group versus 43.8% in the severe disease/ICU group. Patients with newly occurring arrhythmias were at a higher risk of developing severe disease or requiring ICU admission (RR 13.09, 95% CI 7.00 to 24.47, *P* < 0.001; *I*^*2*^ = 42.0%, Fig. 6a). IL-6 levels were significantly higher in the severe disease/ICU group than in the non-severe disease/non-ICU group, as well as in non-survivors than in survivors (SMD 0.54, 95% CI 0.27 to 0.81, *P* < 0.001; *I*^*2*^ = 0.0%, Fig. 6b; SMD 1.28, 95% CI 1.00 to 1.57, *P* < 0.001; *I*^*2*^ = 13.7%, Fig. 6c, respectively).

## Discussion

This systematic review and meta-analysis of 23 high-quality retrospective studies systematically evaluated the risk of severe disease, ICU admission, or death associated with COVID-19-related cardiac injury performance. Our findings are as follows: (1) COVID-19 patients with elevated TnI levels are at significantly higher risk of developing severe disease, requiring ICU admission, or death; (2) elevated CK, CK-MB, LDH, and IL-6 levels and emerging arrhythmia are associated with the development of severe disease or requirement for ICU admission; and (3) mortality rates are significantly higher among patients with elevated LDH and IL-6 levels.

### Cardiac injury

Cardiac injury was defined as a serum cardiac biomarker level (e.g., troponin I) above the 99th percentile upper reference limit or new abnormalities seen on electrocardiography (ECG) and echocardiography [2]. CK, CK-MB, and LDH are also indicators associated with cardiac injury [37, 38]. An elevated cardiac TnI level has high specificity for cardiac injury and is a preferred biomarker of cardiac injury. Overall, in 8 studies including 1028 patients, the rates of elevated TnI or TnT in the non-severe disease/non-ICU admission group and severe disease/ICU admission group were 2.3% and 36.9%, respectively; in the total population, elevated TnI or TnT occurred at a rate of 11.9%. Our analysis suggests that COVID-19 patients with elevated TnI levels are at higher risk of developing severe disease, requiring ICU admission, and death. Two studies from Wuhan (one with 416 cases, another with 187 cases) reported higher mortality among patients with cardiac injury than among those without (51.2% vs. 4.5%; *P* < 0.001; 59.6% vs. 8.9%, *P* < 0.001, respectively) [4, 36]. Patients with cardiac injury had higher serum concentrations of NT-proBNP than those without cardiac injury [36, 39]. Patients with cardiac injury more commonly developed ARDS, were more likely to have ventricular tachycardia (VT) or ventricular fibrillation (VF), and had higher mortality rates than those without VT or VF [36, 39].

TnI has great prognostic significance for patients with COVID-19 as well as those with other influenza virus infections. In a study of 75 inpatients with SARS, acute myocardial infarction was the cause of 2 of 5 deaths [40]. Elevated TnI levels are also common in infections caused by other influenza virus subtypes [41–45]. TnI may play an important role in predicting the acute or long-term risk of influenza virus infection. Other biomarkers closely related to cardiac injury, such as CK, CK-MB, and LDH, were also selected in the meta-analysis. Our analysis showed that those with elevated CK, CK-MB, and LDH were at a higher risk of developing severe disease or requiring ICU admission. The LDH level had a predictive value for death. Previous studies suggested that CK at ICU admission serves can be used as a biomarker of the severity of 2009 pandemic influenza A (pH1N1) infection [46].

Elevated TnI and CK-MB levels indicate cardiac injury such as viral myocarditis or myocardial infarction as well as multiple organ injury [47]. Initial reports showed that the possible pattern of myocardial injury is the early presentation of primary cardiovascular symptoms, as well as changes on echocardiography and ECG [3, 6, 48–50]. Stress cardiomyopathy, supply demand mismatch (type II myocardial infarction), and myocarditis, sometimes similar to ST-segment elevation myocardial infarction, are all possible mechanisms [3, 6, 49]. In a study describing a single case without a history of cardiovascular disease, the patient had myocardial injury, and diffuse edema was seen on cardiac MRI [3]. Twelve lead ECG showed minimal diffuse ST-segment elevation and an ST-segment depression with T-wave inversion of lead V1 and aVR. Even in the absence of respiratory tract or infection symptoms, SARS-CoV-2 infection may cause cardiac involvement. However, it is a pity that an endomyocardial biopsy was not performed; thus, there was no histological evidence [3]. Cardiac injury is an important prognostic factor for COVID-19. It is rational to presume that the virus affects the myocardium, and once patients develop severe pneumonia, cardiac injury or dysfunction is more likely to occur, leading to deterioration. In a study of critically ill patients, including 21 who had SARS-CoV-2 infection in the USA, the incidence of cardiomyopathy was high (*n* = 7 [33%]) [51]. In a patient without fever and respiratory symptoms, the initial ECG showed diffuse ST elevations and an admission TnI level of 7.9 ng/mL, but angiography demonstrated non-obstructive coronary artery disease. After treatment, this patient improved in the short term, but the long-term effects of myocardial injury remain to be determined [8].

The etiology of cardiac dysfunction may be multifactorial and related to infective myocarditis and/or ischemia. Pathological findings suggested a few interstitial mononuclear inflammatory infiltrates in the myocardial interstitial [52]. Viral invasion may cause direct cardiac injury, and COVID-19-induced cytokine storm may also have toxic effects on the myocardium [53]. Cytokine storm may play a role in the development of ARDS and fulminant myocarditis. In our analysis, 3 studies reported the laboratory findings of IL-6 levels in 526 patients. IL-6 levels were significantly higher in the severe disease/ICU groups than non-severe disease/non-ICU groups, as well as in non-survivors than in survivors. Cardiac involvement reportedly occurred a few days after the influenza syndrome, suggesting the mechanisms of a potential myocyte dissemination of the virus activating the immune system, eventually leading to the onset of heart failure [3]. A study reported that, compared to survivors, non-survivors had increased concentrations of C-reactive protein, decreased lymphocyte counts, and significantly reduced numbers of CD3 + CD8 + T cells, resulting in an immune response [54]. Anti-IL-6, as a drug targeting cytokine pathway and based on its mechanism of action, has potential benefits in COVID-19-related ARDS and pneumonia [55].

### Arrhythmia

Our analysis also found that the patients with emerging arrhythmia are at a higher risk of developing severe disease or requiring ICU admission. In a study of 41 patients with COVID-19, atrial fibrillation occurred in 2 of 3 severe and critical patients with tachycardia, with a peak heart rate of 160 bpm [22]. Newly occurring arrhythmias are often closely related to cardiac injury. The incidence of ventricular arrhythmias (VT/VF) among 187 patients with COVID-19 was 5.9%, primarily affecting those with elevated cardiac troponin levels [36]. One study reported that 5 of 6 acute myocardial injury patients had more than two kinds of ECG abnormalities, including ST-T/Q curve abnormality, atrioventricular block, and arrhythmia [28]. Severe pneumonia increases the resistance of the pulmonary circulation, increasing the pressure of the right atrium, and leading to atrial tachyarrhythmia. Antiviral drugs such as hydroxychloroquine may also prolong the QT interval. Alternatively, the virus directly damages the myocardium and the cardiac conduction system, causing multiple ventricular premature and atrioventricular block. More attention is needed on arrhythmia among severe disease/ICU admission COVID-19 patients. However, in the studies reviewed here, ECG or echocardiography was rarely performed and the occurrence of arrhythmia was rarely reported.

## Conclusion

This meta-analysis included the largest sample size and is the first to analyze the correlation of cardiac injury biomarkers and arrhythmia with mortality and other prognosis. Our systematic review and meta-analysis indicate that patients with elevated TnI (TnT) levels are at significantly higher risk of developing severe disease, requiring ICU admission, or death. Our analysis also reveals that patients with elevated CK, CK-MB, and LDH levels and emerging arrhythmia were at a higher risk of developing severe disease, requiring ICU admission. LDH levels also have predictive value for death. Therefore, we strongly recommend the close monitoring of cardiac injury-related biomarkers in COVID-19 patients, especially during the acute disease phase.

### Limitations and prospects

The current clinical attention given to cardiac injury may be insufficient, and the strong infection of the virus makes cardiovascular examinations such as MRI, echocardiography, and coronary angiography difficult to perform [56]. The evaluation of cardiac injury biomarkers combined with cardiac examinations may help better assessments of the condition. There are few reports on cardiac injury in COVID-19 patients, and a large amount of evidence is still needed to make the necessary risk predictions and stratifications. The present results provide some evidence for COVID-19 treatment guidelines. In the future, it well be necessary to strengthen the monitoring of cardiac injury biomarkers, combined with echocardiography [57], ECG, MRI, and other cardiac examinations, in patients with severe SARS-CoV-2 infection. When circulation support is needed in severe COVID-19 cases, the use of an intra-aortic balloon pump or extracorporeal membrane oxygenation may be considered [8].

## Supplementary information

**Additional file 1: Table S1.** Clinical characteristics of patients with COVID-19.

## Data Availability

All the data supporting the conclusions of this article are included within the article.

## Abbreviations

2019-nCoV: Novel coronavirus
ARDS: Acute respiratory distress syndrome
CI: Confidence interval
CK: Creatinine kinase
CK-MB: Creatinine kinase–myocardial band
COVID-19: Coronavirus disease
Hs-TnI: High-sensitivity troponin I
ICU: Intensive care unit
IL-6: Interleukin-6
IQR: Interquartile range
LDH: Lactate dehydrogenase
MC: Multicenter study
*n*: Number
NA: Not available
NCIP: Novel coronavirus-infected pneumonia
NT-proBNP: N-terminal pro-B-type natriuretic peptide
RT-PCR: Reverse transcriptase polymerase chain reaction
SARS-CoV-2: Severe acute respiratory syndrome coronavirus-2
SC: Single-center study
SD: Standard deviation
TnI: Troponin I
TnT: Troponin T
TnT-HSST: Troponin T-hypersensitivity
