# Supplementary material for: Cardiac injury associated with severe disease or ICU admission and death in hospitalized patients with COVID-19: a meta-analysis and systematic review
Source: Crit Care. 2020 Jul 28;24:468. doi: 10.1186/s13054-020-03183-z (PMC7386170; doi:10.1186/s13054-020-03183-z)
Supplement: Supplementary file 1 — Additional file 1: Table S1. Clinical characteristics of patients with COVID-19. [file 13054_2020_3183_MOESM1_ESM.docx]

**Table S1. Clinical characteristics of patients with COVID-19**

|  | **Wang DW et al.** | | **Wu CM et al.(a)** | | **Huang CL et al.** | | **Chen D et al.** | | **Guan WJ et al.** | |
| --- | --- | --- | --- | --- | --- | --- | --- | --- | --- | --- |
| **Characteristic** | **Non-ICU** | **ICU** | **Non-severe disease** | **Severe disease** | **Non-ICU** | **ICU** | **Non-severe disease** | **Severe disease** | **Non-severe disease** | **Severe disease** |
| Elevated TnI/TnT, n/total | 2/102 | 8/36 | NA | NA | 1/28 | 4/13 | NA | NA | NA | NA |
| TnI, Median (IQR), pg/ml | 5.1 (2.1-9.8) | 11 (5.6-26.4) | NA | NA | 3.5 (0.7–5.4) | 3.3 (3–163) | NA | NA | NA | NA |
| Elevated CK, n/total | NA | NA | NA | NA | 7/27 | 6/13 | 13/135 | 11/40 | 67/536 | 23/121 |
| CK, Median (IQR), U/L | 87 (54-121) | 102 (62-252) | NA | NA | 133 (61–189) | 132 (82–493) | NA | NA | NA | NA |
| Elevated CK-MB, n/total | NA | NA | NA | NA | NA | NA | 13/135 | 24/40 | NA | NA |
| CK-MB, Median (IQR), U/L | 18 (12-35) | 13 (10-14) | 15 (12-19) | 17 (13-20.5) | NA | NA | NA | NA | NA | NA |
| Elevated LDH, n/total | NA | NA | NA | NA | 17/27 | 12/13 | 31/135 | 26/40 | 205/551 | 72/124 |
| LDH, Median (IQR), U/L | 212 (171-291) | 435 (302-596) | 257 (211-320.5) | 396 (320-521) | 281 (233–357) | 400 (323–578) | NA | NA | NA | NA |
| Elevated BNP, n/total | NA | NA | NA | NA | NA | NA | NA | NA | NA | NA |
| BNP, Median (IQR), pg/ml | NA | NA | NA | NA | NA | NA | NA | NA | NA | NA |
| Arrhythmia, n/total | 7/102 | 16/36 | NA | NA | NA | NA | NA | NA | NA | NA |
| Elevated IL-6, n/total | NA | NA | NA | NA | NA | NA | NA | NA | NA | NA |
| IL-6, Median (IQR), pg/ml | NA | NA | 6.29 (5.36-7.83) | 7.39 (5.63-10.89) | NA | NA | NA | NA | NA | NA |
| Time of obtaining laboratory parameters | On admission | | From the first day of hospital admission | | On admission | | NA | | On admission | |
| Elevated levels of laboratory parameters | Hs-TnI ≥ 26.2 pg/ml | | NA | | Hs-TnI >28 pg/ml  CK >185 U/L  LDH >245 U/L | | CK >170 U/L  CK-MB >18 U/L  LDH >240 U/L | | LDH ≥ 250 U/L  CK ≥ 200 U/L | |
| Types of arrhythmias | NA | | NA | | NA | | NA | | NA | |

**Table S1. Clinical characteristics of patients with COVID-19** **(continued 1)**

|  | **Hui H et al.** | | **Liu YL et al.** | | **Liu L et al** | | **Qi D et al.** | | **Wang YF et al.** | | **Wu CM et al.(b)** | | **Wu J et al.** | |
| --- | --- | --- | --- | --- | --- | --- | --- | --- | --- | --- | --- | --- | --- | --- |
| **Characteristic** | **Non-severe disease** | **Severe disease** | **Non-severe disease** | **Severe disease** | **Non-severe disease** | **Severe disease** | **Non-severe disease** | **Severe disease** | **Non-severe disease** | **Severe disease** | **Non-ICU** | **ICU** | **Non-severe disease** | **Severe disease** |
| Elevated TnI/TnT, n/total | 0/15 | 4/5 | NA | NA | NA | NA | 0/55 | 3/21 | NA | NA | 35/138 | 27/50 | NA | NA |
| TnI, Median (IQR), pg/ml | NA | NA | NA | NA | NA | NA | NA | NA | NA | NA | NA | NA | NA | NA |
| Elevated CK, n/total | NA | NA | NA | NA | NA | NA | 32/217 | 18/50 | NA | NA | NA | NA | NA | NA |
| CK, Median (IQR), U/L | NA | NA | 100 (53-183) | 83 (49-169) | 59 (41-87) | 42 (35-46) | NA | NA | NA | NA | NA | NA | 67 (52-104) | 76 (41-268) |
| Elevated CK-MB, n/total | NA | NA | NA | NA | NA | NA | 20/217 | 13/50 | NA | NA | 36/138 | 27/50 | NA | NA |
| CK-MB, Median (IQR), U/L | NA | NA | 8.5 (6.0-12.5) | 10.4 (7.0-15.1) | NA | NA | NA | NA | NA | NA | NA | NA | 9(7-14) | 13 (7-24) |
| Elevated LDH, n/total | NA | NA | NA | NA | NA | NA | 39/217 | 18/50 | NA | NA | 27/138 | 35/50 | NA | NA |
| LDH, Median (IQR), U/L | NA | NA | 209 (183-267) | 264 (190-448) | NA | NA | NA | NA | NA | NA | NA | NA | 184 (155-262) | 235 (170-355) |
| Elevated BNP, n/total | NA | NA | NA | NA | NA | NA | NA | NA | NA | NA | NA | NA | NA | NA |
| BNP, Median (IQR), pg/ml | NA | NA | NA | NA | NA | NA | NA | NA | 43.5 (19.0-80.5) | 134.6 (87.25-394.7) | NA | NA | NA | NA |
| Arrhythmia, n/total | 0/11 | 3/6 | NA | NA | NA | NA | NA | NA | NA | NA | NA | NA | NA | NA |
| Elevated IL-6, n/total | NA | NA | NA | NA | NA | NA | 15/29 | 32/38 | NA | NA | NA | NA | NA | NA |
| IL-6, Median (IQR), pg/ml | NA | NA | NA | NA | 0 (0-7.3) | 4.6 (0-28.2) | NA | NA | NA | NA | NA | NA | NA | NA |
| Time of obtaining laboratory parameters | NA | | On admission | | On admission | | On admission | | Severe: on the first, third and seventh day after admission; non-severe: on admission | | On admission | | NA | |
| Elevated levels of laboratory parameters | NA | | NA | | NA | | TnT>14 pg/ml  CK ≥ 170 U/L  CK-MB ≥ 25 U/L  LDH ≥ 245 U/L  IL-6 ≥ 5.3 pg/ml | | BNP ≥ 125 pg/ml | | TnI>28 pg/ml  CK-MB>24 U/L  LDH>250 U/L | | NA | |
| Types of arrhythmias | Atrial fibrillation | | NA | | NA | | NA | | NA | | NA | | NA | |

**Table S1. Clinical characteristics of patients with COVID-19** **(continued 2)**

|  | **Xu HY et al.** | | **Xu YH et al.** | | **Liu YB et al.** | | **Peng YD et al.** | | **Zhang GQ et al.** | | **Liu T et al.** | |
| --- | --- | --- | --- | --- | --- | --- | --- | --- | --- | --- | --- | --- |
| **Characteristic** | **Non-ICU** | **ICU** | **Non-severe disease** | **Severe disease** | **Non-ICU** | **ICU** | **Non-severe disease** | **Severe disease** | **Non-severe disease** | **Severe disease** | **Non-severe disease** | **Severe disease** |
| Elevated TnI/TnT, n/total | 0/45 | 6/8 | NA | NA | 4/265 | 11/26 | NA | NA | 1/166 | 16/55 | NA | NA |
| TnI, Median (IQR), pg/ml | NA | NA | 10 (0-10)  n = 20 | 30 (20-50)  n = 20 | NA | NA | 9.4 (4.75-25.35) | 9.40 (2.30-10.30) | 5.4 (2.2-9.7) | 14.9 (6.9-55.3) | NA | NA |
| Elevated CK, n/total | NA | NA | NA | NA | NA | NA | NA | NA | NA | NA | 0/11 | 10/69 |
| CK, Median (IQR), U/L | NA | NA | 45.0 (30.6-75.4) n = 23 | 83.0 (52.2-221.2) n = 20 | NA | NA | 77.0 (42.0-130.5) | 89.5 (43.5-234.0) | 75 (53-122) | 121 (73-268) | NA | NA |
| Elevated CK-MB, n/total | NA | NA | NA | NA | NA | NA | NA | NA | NA | NA | NA | NA |
| CK-MB, Median (IQR), U/L | NA | NA | NA | NA | NA | NA | 11.0 (9.0-15.0) | 13.0 (8.0-17.0) | 12 (10-15) | 18 (14-35) | NA | NA |
| Elevated LDH, n/total | NA | NA | NA | NA | NA | NA | NA | NA | NA | NA | 1/11 | 45/69 |
| LDH, Median (IQR), U/L | NA | NA | 285.3 (215.5-346.7) | 397.1 (342.2-523.8) | NA | NA | 290.0 (227.5-372.5) | 351.0 (239.0-413.5) | 204 (167-290) | 424 (287-591) | NA | NA |
| Elevated BNP, n/total | NA | NA | NA | NA | NA | NA | NA | NA | NA | NA | NA | NA |
| BNP, Median (IQR), pg/ml | NA | NA | NA | NA | NA | NA | 33.4 (21.9-75.8) | 20.35 (10.0-77.05) | NA | NA | NA | NA |
| Arrhythmia, n/total | 1/45 | 5/8 | NA | NA | NA | NA | NA | NA | 2/166 | 22/55 | NA | NA |
| Elevated IL-6, n/total | NA | NA | NA | NA | NA | NA | NA | NA | NA | NA | NA | NA |
| IL-6, Median (IQR), pg/ml | NA | NA | NA | NA | NA | NA | NA | NA | NA | NA | NA | NA |
| Time of obtaining laboratory parameters | On admission | | NA | | On admission | | On admission | | On admission | | Laboratory findings were collected on admission | |
| Elevated levels of laboratory parameters | TnT ≥ 28 pg/ml | | NA | | TnI>0.03 μg/L | | NA | | NA | | LDH ≥ 250 U/L  CK ≥ 200 U/L | |
| Types of arrhythmias | Tachycardia and atrioventricular block | | NA | | NA | | NA | | NA | | NA | |

**Table S1. Clinical characteristics of patients with COVID-19** **(continued 3)**

|  | **Wu CM et al.(a)** | | **Yang XB et al.** | | **Fu L et al.** | | **Chen T et al.** | | **Guo T et al.** | |
| --- | --- | --- | --- | --- | --- | --- | --- | --- | --- | --- |
| **Characteristic** | **Survivors** | **Non-survivors** | **Survivors** | **Non-survivors** | **Survivors** | **Non-survivors** | **Survivors** | **Non-survivors** | **Survivors** | **Non-survivors** |
| Elevated TnI/TnT, n/total | NA | NA | 3/20 | 9/32 | 112/166 | 25/34 | 15/109 | 68/94 | 21/144 | 31/43 |
| TnI, Median (IQR), ug/ml | NA | NA | NA | NA | NA | NA | 3.3 (1.9-7.0) n = 109 | 40.8 (14.7-157.8) n = 94 | NA | NA |
| Elevated CK, n/total | NA | NA | NA | NA | NA | NA | NA | NA | NA | NA |
| CK, Median (IQR), U/L | NA | NA | NA | NA | 79.5 (50.0-144.5) | 180.5 (80.8-404.5) | 84.0 (50.8-140.3) | 189.0 (94.5-374.5) | NA | NA |
| Elevated CK-MB, n/total | NA | NA | NA | NA | NA | NA | NA | NA | NA | NA |
| CK-MB, Median (IQR), U/L | 16 (13-20.75) | 17 (13-20) | NA | NA | NA | NA | NA | NA | NA | NA |
| Elevated LDH, n/total | NA | NA | NA | NA | NA | NA | NA | NA | NA | NA |
| LDH, Median (IQR), U/L | 349.5 (293.5-416) | 484 (351-568.5) | NA | NA | 245.0 (179.0-345.0) | 495.5 (350.8-695.8) | 268.0 (214.3-316.5) | 564.5 (431.0-715.8) | NA | NA |
| Elevated BNP, n/total | NA | NA | NA | NA | NA | NA | NA | NA | NA | NA |
| BNP, Median (IQR), pg/ml | NA | NA | NA | NA | NA | NA | 72.0 (20.0-185.0) n = 93 | 800.0 (389.8-1817.5) n=80 | NA | NA |
| Arrhythmia, n/total | NA | NA | NA | NA | NA | NA | NA | NA | NA | NA |
| Elevated IL-6, n/total | NA | NA | NA | NA | NA | NA | NA | NA | NA | NA |
| IL-6, Median (IQR), pg/ml | 6.05 (5.12-6.99) | 10.07 (7.36-14.8) | NA | NA | NA | NA | 13.0 (4.0-26.2) n = 110 | 72.0 (35.6-146.8) n = 53 | NA | NA |
| Time of obtaining laboratory parameters | On admission | | On admission | | NA | | On admission | | On admission | |
| Elevated levels of laboratory parameters | NA | | NA | | CK＞174 U/L | | TnI＞15.6 pg/mL | | NA | |
| Types of arrhythmias | NA | | NA | | NA | | NA | | NA | |

*BNP* B-type natriuretic peptide, *CK* creatinine kinase, *CK-MB* creatinine kinase–myocardial band, *Hs-TnI* high-sensitivity troponin I, *ICU* intensive care unit, *LDH* lactate dehydrogenase, *IL-6* interleukin-6, *IQR* interquartile range, *n* number, *NA* not available, *Tn*I troponin I, *TnT* troponin T.
